# Non-Alcoholic Fatty Liver in Patients with Chylomicronemia

**DOI:** 10.3390/jcm10040669

**Published:** 2021-02-09

**Authors:** Mélanie Maltais, Diane Brisson, Daniel Gaudet

**Affiliations:** Lipidology Unit, Community Genomic Medicine Center, Department of Medicine, Université de Montréal, ECOGENE-21 Clinical and Translational Research Center, Chicoutimi, QC G7H 7K9, Canada; melanie.maltais@umontreal.ca (M.M.); diane.brisson@ecogene21.org (D.B.)

**Keywords:** non-alcoholic fatty liver disease, obesity, triglyceride, familial chylomicronemia syndrome, multifactorial chylomicronemia, pancreatitis, transient elastography

## Abstract

Non-alcoholic fatty liver disease (NAFLD) is frequent in patients with features of the metabolic syndrome (MetS), obesity, or type 2 diabetes. Lipoprotein lipase (LPL) is the main driver of triglyceride (TG) hydrolysis in chylomicrons and very-low density lipoproteins (VLDL). In some patients with MetS, dysfunction of this pathway can lead to plasma TG values > 10 mmol/L (multifactorial chylomicronemia or MCS). Chylomicronemia also characterizes LPL deficiency (LPLD), a rare autosomal recessive disease called familial chylomicronemia syndrome (FCS), which is associated with an increased risk of recurrent pancreatitis. This study aims to investigate the expression of NAFLD, as assessed by transient elastography, in MCS and FCS subjects. Data were obtained from 38 subjects with chylomicronemia; 19 genetically confirmed FCS and 19 sex- and age-matched MCS. All participants underwent liver ultrasonography and stiffness measurement after a 4-h fast using transient elastography (FibroScan^®^, Echosens, Waltham, MA, USA). NAFLD (controlled attenuation parameter (CAP) > 280 dB/m) was observed in 42.1% of FCS and 73.7% of MCS subjects (*p* = 0.05). FCS subjects had lower body mass index (BMI) than MCS. Only 25% of FCS subjects with NAFLD had a BMI ≥ 30 compared to 64.3% in MCS (*p* = 0.004). In FCS, NAFLD occurred even in the presence of very low (≤18 kg/m^2^) BMI. In both FCS and MCS, CAP was negatively associated with acute pancreatitis risk. In this study, NAFLD was commonly observed in both FCS and MCS subjects and occurred independently of the BMI and fasting glucose values in FCS; NAFLD was associated with a lower occurrence of acute pancreatitis episodes.

## 1. Introduction

Non-alcoholic fatty liver disease (NAFLD) is a chronic liver disease characterized by an excessive fat accumulation in the liver, in absence of secondary causes, such as important alcohol consumption, viral hepatitis, medication or autoimmune diseases. NAFLD can progress from simple steatosis (non-alcoholic fatty liver, NAFL) toward non-alcoholic steatohepatitis (NASH). NASH is characterized by the presence of lobular inflammation, with or without fibrosis, and can ultimately lead to cirrhosis or liver cancer [1,2]. NAFLD is by far the most frequent liver disease, with an estimated prevalence of 25% worldwide. It is frequently observed in patients presenting features of the metabolic syndrome (MetS): type 2 diabetes, insulin resistance, hypertriglyceridemia, and visceral obesity. Its expression is therefore enhanced by some environment factors, such as poor eating behaviors and lack of exercise [3,4,5]. NAFLD is associated with increased risks of cardiovascular disease and diabetes mellitus in non-diabetic patients. NAFLD is a consequence of triglyceride (TG) dysmetabolism and signaling defects involving TG intermediates, among which fatty acids (FA) and diacylglycerol. Around 60% of FA delivered to the liver originates from the white adipose tissue. Chylomicrons, the large TG-rich lipoproteins produced by the gut following a meal, play an important role in delivering FA to adipose tissue and TG to the liver. In the circulation, TG carried in chylomicrons are metabolized by the key enzyme lipoprotein lipase (LPL), releasing free FA, which are subsequently metabolized by peripheral (muscle and adipose) tissue, and chylomicron remnants are formed. Chylomicron remnants and their residual TG content are then taken up by the liver [6,7,8].

In some patients with MetS, dysfunction of this pathway can lead to TG concentrations >10 mmol/L and plasma accumulation of chylomicrons, a condition called chylomicronemia. Multifactorial chylomicronemia (MCS) is a polygenic disorder frequently associated with obesity and other elements of the MetS, in addition to unhealthy life habits. The estimated prevalence of MCS is approximately 160–170 per 100,000 individuals in the occidental world [9]. Chylomicronemia also characterizes LPL deficiency (LPLD), a rare autosomal recessive disease (0.1–0.2 per 100,000 individuals) called familial chylomicronemia syndrome (FCS) [10]. FCS is not associated with obesity, most patients being of normal or even underweight, including some with signs of lipoatrophy. This disorder is caused by null loss-of-function variants in the LPL gene or in genes directly affecting LPL availability or activity: apolipoprotein C2 (APOC2), apolipoprotein A5 (APOA5), lipase maturation factor 1 (LMF1), or glycosylphosphatidylinositol-anchored high-density lipoprotein-binding protein 1 (GPIHBP1) [9,10].

An increasing number of studies highlight clinically important differences between MCS and FCS phenotypes in term of clinical expression, risk of disease, drug response, and disease management [11,12,13,14,15,16,17]. Both FCS and MCS are associated with an increased acute pancreatitis risk but the risk is the highest in FCS patients. Very little is known about the expression of NAFLD in presence of chylomicronemia. Therefore, the objective of this study is to investigate the expression of NAFLD, as assessed by transient elastography, in patients with FCS and MCS.

## 2. Materials and Methods

### 2.1. Subjects

This cross-sectional study was performed in a sample of 38 chylomicronemic (TG > 10 mmol/L) French Canadian adults: 19 FCS sex- and age- (±10 years) matched to 19 MCS individuals. Patients were classified as FCS when they presented sustained chylomicronemia, were either homozygotes or compound heterozygotes for null, FCS-causing, loss-of-function mutations in the LPL gene, presented low (<5%) post-heparin LPL activity, and reached the FCS diagnosis score threshold using both European and Canadian diagnosis scoring systems [11,18,19]. Otherwise a participant was classified as MCS. More precisely, MCS patients did not reach the threshold of FCS diagnosis while using clinical diagnosis systems. In addition, all MCS patients in this study, were genotyped for FCS-causing mutations explaining almost all cases in the French Canadian founder population [12] and none were homozygotes or compound heterozygotes for variants known to cause FCS in this population. In addition, gene sequencing of FCS-causing genes (LPL, APOC2, APOA5, GPIHBP1, or LMF1) is systematically performed at our clinic among ambiguous cases. No subject had an abusive alcohol consumption, as defined by the consumption of ≥20 g/day or ≥30 g/day for women and men, respectively, or any other secondary causes of NAFLD, such as viral hepatitis, medication, or autoimmune diseases [20]. Obesity was defined by a body mass index (BMI) ≥ 30 kg/m^2^. Type 2 diabetes was diagnosed according to Diabetes Canada and the World Health Organization criteria as fasting glucose > 7.0 mmol/L measured twice, random glycemia > 11.1 mmol/L or a 2 h glucose concentration > 11.1 mmol/L following a 75g oral glucose load. History of pancreatitis was documented using questionnaires and subject’s medical charts, applying the Atlanta classification criteria [21]. Recurrent acute pancreatitis (RAP) was defined as the past occurrence of ≥4 confirmed acute pancreatitis episodes. Subjects gave their informed consent to participate in this study and were assigned a code that systematically de-identifies all clinical data [22]. This study was conducted in accordance with the Declaration of Helsinki and was approved by the IRB Services (now Advarra, Approval Number: Pro00013642).

### 2.2. Blood Samples

Blood samples were obtained after a 12-h overnight fast. Cholesterol, TG, and glucose levels were enzymatically measured on a CX7 Analyzer (Beckman, Fullerton, CA, USA). The high-density lipoprotein (HDL) subfraction was obtained after precipitation of low-density lipoproteins (LDL) (d > 1.006 g/mL) in the infranatant with heparin and MnCl_2_. Alanine aminotransferase (ALT) was analyzed using Beckman uniCel DxC800 Synchron (Beckman, Fullerton, CA, USA).

### 2.3. Transient Elastography Analysis

Transient elastography was performed by a certified operator among subjects who had fasted for at least 3 h [23] to measure the degree of ultrasound attenuation by hepatic fat (controlled attenuation parameter (CAP)) and liver stiffness (liver stiffness measurement (LSM)) using FibroScan^®^ (Echosens, Waltham, MA, USA). The M or XL probes were used according to the subject’s size. CAP and LSM results were expressed in dB/m and kPa, respectively. Results with ten valid measurements and interquartile range/median LSM ratio < 30% were considered. Patients were considered as presenting NAFLD when CAP score was above 280 dB/m and with liver stiffness when LSM ≥ 7 kPa [24]. Examination results were assessed by a physician with experience in FibroScan^®^ results.

### 2.4. Statistical Analysis

Categorical variables were compared using the Pearson χ^2^ or Fisher’s exact tests, whereas continuous variables were compared with two-sided Student t-test, one-way ANOVA, using log_10_-transformed data, followed by Bonferroni post-hoc tests, or Kruskal–Wallis analyses and Mann–Whitney U-tests for variables staying non-normally distributed after transformation. All reported *p* values were assessed at the 5% level. Data were analyzed using SPSS package (IBM SPSS Statistics, Version 25.0. Armonk, NY, USA).

## 3. Results

Table 1 shows FCS and MCS subjects’ characteristics. There were 42.1% of males, and mean age is 53.1 ± 17.9 years in FCS and 56.7 ± 13.8 years in MCS. The FCS group presented lower HDL-cholesterol, CAP score, and BMI than MCS. Obesity (BMI ≥ 30 kg/m^2^) and overweight (BMI ≥ 25 kg/m^2^) were significantly more prevalent in MCS whereas a FCS patient was underweighted (BMI ≤ 18 kg/m^2^). As compared to MCS, RAP was more prevalent in FCS than in MCS (84.2% vs. 10.5%; *p* < 0.001). FCS subjects also tend to present higher total TG levels (28.5 (22.0–45.0) mmo/L vs. 18.0 (12.9–33.0) mmol/L; *p* = 0.1) than MCS. Type 2 diabetes was observed in 42.1% (*n* = 8) of FCS and 57.9% (*n* = 11) of MCS respectively. No cases of diabetes due to pancreatic insufficiency consecutive to recurrent acute pancreatic were observed. Half (*n* = 4) of FCS diabetic patients were controlled with the diet alone, two received oral anti-diabetic agent, whereas two required insulin therapy. In comparison, none of the MCS diabetic patients were treated with the diet alone and 45.5% (*n* = 5) required insulin therapy. At the moment of the study, no diabetic participants were receiving any oral anti-diabetic agent known to affect acute pancreatitis risk or fatty liver (such as GLP-1 agonists or SGLT-2 inhibitors). Glycated hemoglobin (HbA1c) data suggest that diabetes was fairly well controlled in both groups.

Stratifying chylomicronemic patients according to their FCS or MCS status and CAP score (Table 2) highlighted significant differences in NAFLD expression. A majority (64.3%) of MCS subjects with NAFLD (CAP ≥ 280 dB/m) were obese and 78.6% had a BMI ≥ 25 kg/m^2^ (overweight or obese), compared to 25% and 37.5%, respectively, in FCS (*p* = 0.001). Besides, 63.6% of FCS subjects with CAP score < 280 dB/m had diabetes, while diabetes affected only 12.5% of FCS subjects with NAFLD. All FCS patients of this study had at least one prior episode of acute pancreatitis. In MCS however, only 21.4% of patients with NAFLD had a prior history of acute pancreatitis, compared to 60% of patients with a normal CAP score. A CAP score > 280 dB/m was negatively associated with the RAP in both FCS and MCS groups (*p* < 0.001). However, the correlation between the CAP score and the number of pancreatitis episodes is significant only among FCS (r_s_ = −0.64, *p* = 0.003).

**Figure 1:**
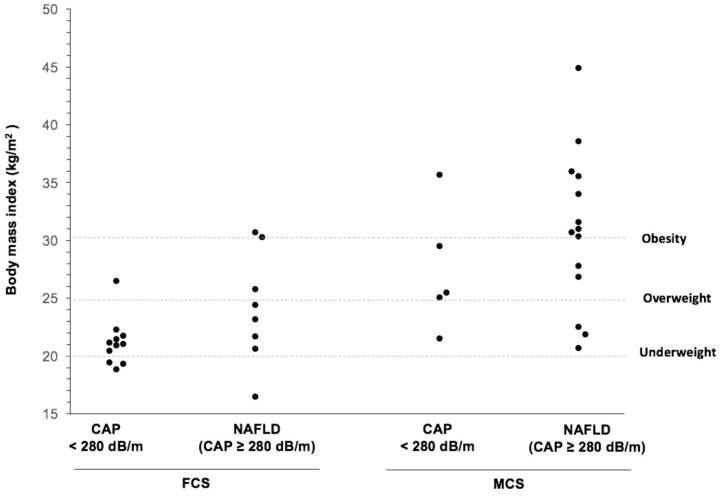
Body mass index in FCS and MCS subjects, according to CAP score. CAP, controlled attenuation parameter; FCS, familial chylomicronemia syndrome; MCS, multifactorial chylomicronemia; NAFLD, non-alcoholic fatty liver disease.

The BMI and the CAP score are positively correlated in both FCS (r_s_ = 0.59; *p* = 0.008) and MCS (r_s_ = 0.41; *p* = 0.08) (data not shown). However, as shown in Figure 1, in contrast to MCS, only two FCS subjects had a BMI ≥ 30 kg/m^2^ (30.3 and 30.7 kg/m^2^ respectively) and four were either obese or overweight (BMI ≥ 25 kg/m^2^). NAFLD in FCS was observed even in an underweighted patient (BMI ≤ 18 kg/m^2^).

## 4. Discussion

With the world endemization of obesity, sedentary lifestyle, and diet westernization, approximately 25% of adults in the occidental world might exhibit NAFLD, by far the most common liver disease worldwide [4]. Results of the present study suggest that NAFLD could be a highly significant clinical feature of chylomicronemia since 42% of FCS (inherited LPL deficiency) and 74% of MCS (polygenic or functional LPL deficiency) patients presented criteria of NAFLD, as assessed by transient elastography. Prevalence of liver stiffness, also higher among MCS and FCS than in the general population, would be comparable in both groups. In a meta-analysis involving over 8 million individuals from 22 countries, more than 80% of NAFLD patients were either overweight or obese, 72% had hypertriglyceridemia, and 44% had type 2 diabetes mellitus [25]. Therefore, NAFLD is now considered as an hepatic clinical component or consequence of the MetS.

The most common form of chylomicronemia, MCS, is associated with the classical features of the MetS, which is not the case for FCS [11,12,13,14,15]. As observed, compared to MCS, FCS patients are in general non obese and rarely overweight and NAFLD (CAP score > 280 dB/m) was regularly observed in normal weight and underweight FCS patients.

The close association between NAFLD and the BMI has been documented by so many studies that it is not surprising to see a significant correlation between these variables in MCS patients of the present study since a positive energy balance is a trigger of TG storage [25,26,27,28]. A disturbed regional mobilization of TG and FA, associated with insulin signaling and metabolism alterations, which are frequently observed in obese patients with NAFLD, will then promote the storage of excess calories, mainly TG, in hepatocytes. This hepatic accumulation of lipids will first lead to simple steatosis (NAFL) and eventually to NASH if not timely managed [28,29,30]. Obesity and other components of the MetS, including type 2 diabetes and insulin resistance, are important contributors to chylomicronemia in MCS but not in FCS [11,12,13,14,15].

Although less frequent than in MCS, NAFLD is not rare in FCS and results of the current study suggest that it could be more prevalent in both disorders than in the general population. NAFLD is regularly observed in patients with obesity and other elements of the metabolic syndrome. In contrast to MCS, most FCS patients are not overweight and NAFLD is observed in FCS patients with normal (<25 kg/m^2^) or low BMI (≤18 kg/m^2^). It has been reported that prevalence of NAFLD among the non-obese population might range between 10% and 30% [31]. It appears that visceral fat and insulin resistance could be important determinants of NAFLD among lean individuals [32]. FCS is a complex phenotype associated with acylglycerol dysmetabolism due to LPL deficiency affecting fatty acid signaling pathways, glucose homeostasis, VLDL production, and peripheral fat management. Physiopathological mechanisms of NAFLD among non-obese individuals are not yet fully understood but published data suggest that the lean-NAFLD phenotype could be a different entity than obesity-related NAFLD [33].

A pivotal step in NAFLD onset is TG accumulation into the liver cells [7]. TG are synthesized from FA and free glycerol. FA from the white adipose tissue is usually delivered to the hepatocytes or produced from glucose via lipogenesis. FA are catabolized primarily by β-oxidation and are subsequently available for monoacylglycerol, diacylglycerol, and TG synthesis. A significant proportion of TG are also directly delivered to the liver by chylomicron remnants. Once delivered or synthesized, TG are stored as lipid droplets in the hepatocytes and used to construct very-low-density lipoprotein (VLDL) particles that are eventually secreted in the circulation. Disruption in signaling parameters or any metabolic step of intra-hepatic TG-related pathways or adipocyte FA mobilization or overflow can result in hepatosteatosis [6,7]. Hepatocytes thus store extra lipids when the storing capacity of adipose tissue is insufficient or is dysfunctional, a situation which characterized by the MetS as well as lipodystrophy. Lipodystrophy is a group of adipose tissue disorders characterized by a loss of subcutaneous adipose tissue that can result in the ectopic storage of lipid and metabolic perturbations, including NAFLD [29,34]. NAFLD is indeed a frequent feature of partial lipodystrophy [35], which indicates the importance of the liver-adipose tissue communication axis for the development of NAFLD. FCS is by nature a genetically determined model of partial lipodystrophy, covering a wide range of phenotypes including underweight patients presenting abnormally low skin folds an almost complete absence of subcutaneous adipose tissue [36]. Partial or complete lipodystrophy has also been associated with an increased pancreatic fat content [37], thus conferring a pro-inflammatory environment at the pancreas immediate vicinity. Accordingly, intrapancreatic fat (non-alcoholic fatty pancreas disease, NAFPD) has been identified as an independent correlate of acute pancreatitis [38]. NAFPD, defined as an excessive lipid accumulation within the pancreas in the absence of alcohol intake, appears to show several similarities with NAFLD, both being associated with obesity and other elements of the MetS (insulin resistance, high serum lipid levels, and high blood pressure). However, consequences of NAFPD are far less understood than those of NAFLD [39,40]. Results of this study suggest that there is a negative correlation between liver fat accumulation and acute pancreatitis risk in presence of chylomicronemia, suggesting that if more TG accumulates in the liver (NAFLD), less is available to contribute to the pancreatic fat pad (NAFPD) and associated pro-inflammatory environment, while the opposite could be observed for other patients. Gene expression signatures and clinical profiles of pancreatitis risk among chylomicronemic patients have been recently identified and several differentially expressed genes are associated with inflammation and fat metabolism [41]. Stratification for NAFLD and body fat distribution for these gene and clinical signatures are currently ongoing by using dynamic positron emission tomography (PET) technology to assess the body fat distribution in patients with chylomicronemia with or without RAP.

Various factors were identified as independent correlates of steatosis severity and CAP score in previous studies, including but not limited to elevated levels of total cholesterol, TG, and blood glucose [42,43]. In the current study, univariate analyses did not reveal significant correlations between CAP and these metabolic parameters, including plasma TG, in both MCS and FCS patients. The small size of our sample can be of course partly responsible for this result. In addition, the apparently surprising absence of correlation between plasma TG and CAP could also be explained by the fact that the range of triglyceride levels only encompasses very high concentrations.

Given the rarity of FCS, one of the strength of this study was the inclusion of nearly 20 finely phenotyped patients age and gender-matched with MCS patients issued from a founder population [44]. This study has also limitations. The sample size remains small despite the rarity of FCS, which is clearly a significant weakness of the current study, and results must be replicated in a larger and more diversified sample. Also, transient elastography (FibroScan^®^) although a validated technique, remains a surrogate of NAFLD assessment compared to liver biopsy, which is however invasive.

## 5. Conclusions

In conclusion, this cross-sectional study suggests that hepatic steatosis is a significant clinical component of chylomicronemia, differentially expressed in FCS and MCS. Obtained results therefore agree that individual risks and disease management are not the same in FCS and MCS. While MCS patients with overweight are exposed to a higher risk of NAFLD, current observation suggest that clinicians should not base their investigation of NAFLD on BMI among FCS patients. The negative correlation between accumulation of liver fat (NAFLD) and the occurrence of acute pancreatitis episodes in patients with chylomicronemia observed in this study requires further investigation. Such studies are ongoing.

## Figures and Tables

**Table 1:** Subject characteristics.

	FCS (*n* = 19)	MCS (*n* = 19)	*p*-Value
Male, *n* (%)	8 (42.1)	8 (42.1)	NS
Age (years)	53.1 ± 17.9	56.7 ± 13.8	NS
BMI (kg/m^2^)	22.4 ± 3.7	30.0 ± 6.5	<0.001
BMI ≥ 30 kg/m^2^, *n* (%)	2 (10.5)	10 (52.6)	0.005
BMI ≥ 25 kg/m^2^, *n* (%)	4 (21.1)	15 (78.9)	<0.001
Type 2 diabetes, *n* (%)	8 (42.1)	11 (57.9)	NS
History of pancreatitis, *n* (%)	19 (100)	6 (31.6)	<0.001
Recurrent acute pancreatitis, *n* (%)	16 (84.2)	2 (10.5)	<0.001
ALT (U/L)	18.0 ± 11.3	29.8 ± 22.3	0.05
Fasting glucose (mmol/L)	5.35 ± 0.74	5.98 ± 2.03	NS
HbA1c (%) ^*^	6.6 (6.2–6.6)	7.8 (6.7–8.3)	NS
HDL cholesterol (mmol/L)	0.37 ± 0.17	0.60 ± 0.25	0.06
Total triglycerides (mmol/L) ^*^	28.5 (22.0–45.0)	18.0 (12.9–33.0)	0.1
CAP (dB/m)	276 ± 50	322 ± 54	0.01
LSM (kPa) ^*^	5.1 (4.1–6.5)	5.9 (5.1–7.7)	NS
NAFLD (CAP > 280dB/m), *n* (%)	8 (42.1)	14 (73.7)	0.05
Liver stiffness, (LSM ≥ 7 kPa) *n* (%)	3 (15.8)	5 (26.3)	NS

Mean ± s.d. unless otherwise specified. ^*^ Median (Interquartile range). ALT, alanine aminotransferase; BMI, body mass index; CAP, controlled attenuation parameter; FCS, familial chylomicronemia syndrome; HbA1c, hemoglobin A1c (glycated hemoglobin); HDL, high-density lipoprotein; LSM, liver stiffness measurement; MCS, multifactorial chylomicronemia; NAFLD, non-alcoholic fatty liver disease. HbA1c was available only for patients with diabetes. NS: *p* > 0.1.

**Table 2:** Comparison of FCS and MCS subjects according to the CAP score.

	FCS		MCS		*p*-Value
	CAP < 280 (a) *n* = 11	CAP ≥ 280 (b) *n* = 8	CAP < 280 (c) *n* = 5	CAP ≥ 280 (d) *n* = 14	
Male, *n* (%)	5 (45.5)	3 (37.5)	1 (20)	7 (50)	NS
Age (years)	48.8 ± 19.9	59.0 ± 13.8	51.6 ± 17.0	58.5 ± 12.7	NS
BMI (kg/m^2^)	21.2 ± 2.1	24.2 ± 4.8	27.4 ± 5.4	30.9 ± 6.8 ^a,b^	<0.001
BMI ≥ 30 kg/m^2^, *n* (%)	0 (0)	2 (25.0)	1 (20)	9 (64.3)	0.004
BMI ≥ 25 kg/m^2^, *n* (%)	1 (9.1)	3 (37.5)	4 (80)	11 (78.6)	0.001
Type 2 diabetes, *n* (%)	7 (63.6)	1 (12.5)	3 (60)	8 (57.1)	NS
History of pancreatitis, *n* (%)	11 (100)	8 (100)	3 (60)	3 (21.4)	<0.001
Recurrent acute pancreatitis, *n* (%)	11 (100)	5 (62.5)	1 (20)	1 (7.1)	<0.001
ALT (U/L)	14.9 ± 5.6	22.1 ± 15.8	16.0 ± 4.8	33.8 ± 23.8	0.048
Fasting glucose (mmol/L)	5.47 ± 0.86	5.17 ± 0.52	6.57 ± 3.63	5.80 ± 1.44	NS
HDL cholesterol (mmol/L)	0.33 ± 0.06	0.43 ± 0.31	0.44 ± 0.28	0.67 ± 0.22	NS
Total triglycerides (mmol/L) ^*^	30.3 (19.1–44.7)	28.5 (22.5–47.3)	28.1 (9.6–87.8)	17.5 (12.8–28.9)	NS
CAP (dB/m)	241 ± 31	324 ± 26 ^a^	251 ± 21 ^b^	347 ± 35 ^a,c^	<0.001
LSM (kPa) ^*^	4.9 (3.7–6.3)	5.7 (4.9–8.5)	5.9 (5.1–7.1)	5.9 (5.0–8.0)	NS

Mean ± s.d. unless otherwise specified. ^*^ Median (Interquartile range). ALT, alanine aminotransferase; BMI, body mass index; CAP, controlled attenuation parameter; FCS, familial chylomicronemia syndrome; HDL, high-density lipoprotein; LSM, liver stiffness measurement; MCS, multifactorial chylomicronemia. Significantly different (*p* < 0.05) as compared to ^a^ FCS with CAP < 280, ^b^ FCS with CAP ≥ 280, ^c^ MCS with CAP < 280, following Bonferonni correction for multiple testing. Spearman’rank correlation (rs) between CAP and acute pancreatitis risk (number of pancreatitis): rs = −0.64, *p* = 0.003 among FCS and rs = −0.15, NS among MCS. NS: *p* > 0.1.

## Data Availability

The datasets used and/or analyzed during the current study are available from the corresponding author on reasonable request.
